# Feasibility, acceptability, and bacterial recovery for community-based sample collection to estimate antibiotic resistance in commensal gut and upper respiratory tract bacteria

**DOI:** 10.1038/s41598-022-27084-z

**Published:** 2022-12-29

**Authors:** Hoang Huy Tran, Hien Anh Thi Nguyen, Huyen Bang Tran, Bich Ngoc Thi Vu, Tu Cam Thi Nguyen, Costanza Tacoli, Thao Phuong Tran, Tung Son Trinh, Thien Huong Ngoc Cai, Behzad Nadjm, Kieu Hương Thi Tran, Quynh Dieu Pham, Thương Hong Thi Nguyen, Trang Thu Nguyen, Thai Duy Pham, Thomas Kesteman, Duc Anh Dang, Tien Dac Tran, H. Rogier van Doorn, Sonia Lewycka

**Affiliations:** 1 National Institute for Hygiene and Epidemiology, Hanoi, Vietnam; 2 Oxford University Clinical Research Unit, Hanoi, Vietnam; 3 Medical Research Council Unit The Gambia at the London, School of Hygiene and Tropical Medicine, Serekunda, The Gambia; 4 Centre for Tropical Medicine and Global Health, Nuffield Department of Medicine, University of Oxford, Oxford, UK; 5 Centre for Disease Control, Phu Ly, Ha Nam Province Vietnam

**Keywords:** Applied microbiology, Epidemiology

## Abstract

Vietnam has high rates of antibiotic use and resistance. Measuring resistance in commensal bacteria could provide an objective indicator for evaluating the impact of interventions to reduce antibiotic use and resistance. This study aimed to evaluate the feasibility, acceptability, and bacterial recovery for different sampling strategies. We conducted a cross-sectional mixed methods study in a rural community in Ha Nam Province, northern Vietnam, and collected structured interviews, samples, and in-depth interviews from households. Out of 389 households invited, 324 participated (83%), representing 1502 individuals. Samples were collected from these individuals (1498 stool, 1002 self-administered nasal swabs, and 496 HW-administered nasopharyngeal swabs). Pneumococci were recovered from 11.1% (128/1149) of the total population and 26.2% (48/183) of those under 5-years. Recovery was higher for health-worker (HW)-administered swabs (13.7%, 48/350) than self-administered swabs (10.0%, 80/799) (OR 2.06, 95% CI 1.07–3.96). Cost per swab was cheaper for self-administered ($7.26) than HW-administered ($8.63) swabs, but the overall cost for 100 positive samples was higher ($7260 and $6300 respectively).﻿ Qualitative interviews revealed that HW-administered nasopharyngeal swabs took longer to collect, caused more discomfort, and were more difficult to take from children. Factors affecting participation included sense of contribution, perceived trade-offs between benefits and effort, and peer influence. Reluctance was related to stool sampling and negative perceptions of research. This study provides important evidence for planning community-based carriage studies, including cost, logistics, and acceptability. Self-administered swabs had lower recovery, and though cheaper and quicker, this would translate to higher costs for large population-based studies. Recovery might be improved by swab-type, transport medium, and better cold-chain to lab.

## Introduction

Antibiotic resistance is a global public health problem. Vietnam has antibiotic resistance rates amongst the highest in the world, driven by limited antibiotic stewardship, proliferation and poor regulation of private pharmacies, and self-medication due to common social beliefs of the role of antibiotics in treatment of illness^1^. Vietnam’s National Action Plan on combatting drug resistance mainly focused on surveillance and antimicrobial stewardship in hospital-based settings^2,3^. However, the volume of antibiotics consumed in primary healthcare and community settings is much higher and has received less attention^1^. Interventions targeting primary healthcare settings and the public have been successful in high income countries^4–6^, but there have been few population-level interventions to tackle inappropriate antibiotic use and resistance in low- and middle-income countries.

The impact of population-based interventions is typically measured in terms of antibiotic prescriptions or consumption^7^, but these data are difficult to collect reliably in settings with limited electronic health records. Furthermore, prescription courses of antibiotics account for a small proportion of those consumed in the community in many low- and middle-income countries (LMIC), and antibiotics may be purchased from many licensed and unlicensed sources^8,9^. Asking community members about antibiotics consumed may also be inaccurate, as many people consume pills without knowing what they are^10^. Finally, reductions in antibiotic consumption can be considered a proxy measure, where the true metric of interest is the prevalence of antibiotic resistance in the community. In order to provide an objective measure of the impact of public health policies and interventions on the problem of inappropriate antibiotic use and resistance, we propose that measuring resistance patterns in commensal bacteria may provide a more robust and comparable indicator. Resistance among commensal flora has been shown to be sensitive to intervention^11–13^. We chose to focus on *Streptococcus pneumoniae* and *Enterobacterales* because they represent Gram positive and Gram negative bacteria from different microbiota. These species were chosen as indicator species that could be affected by individual and population-level changes in antibiotic use. Carriage of *S. pneumoniae* has been successfully used as a microbiological endpoint in a community trial before^14^, and is also of interest because of the potential to reduce colonisation with resistant strains through vaccines^15^.

*S. pneumoniae* are part of the normal bacterial flora in the human upper respiratory tract. Carriage of *S. pneumoniae* is generally higher among children than adults, with the highest prevalence found among children under 5-years old^16^. A systematic review and meta-analysis reported carriage among healthy children under 5-years old ranging from 20 to 93.4% in low-income countries (pooled prevalence 64.8%)and from 6.5 to 69.8% in lower-middle income countries (pooled prevalence 47.8%)^16^. Carriage in children 5-years and over and adults was not as widely studied, but combined estimates ranged from 29.1 to 86.3% and 7.3–60.6% respectively among healthy populations in low- and lower-middle income countries^16^. In Vietnam, a lower-middle income country, estimates of carriage among children under 5-years have ranged from 28.7 to 52% depending on the age composition of the participants, sample method, time period, and location^8,17–24^. There was only one study reporting carriage among the general population (11%), including children under 5-years (43%), 5–18 years (12%), and adults (2%)^17^.

The WHO Pneumococcal Carriage Working group has developed recommendations for methods for detecting upper respiratory carriage of *S. pneumoniae*. A single nasopharyngeal (NP) swab administered by health-workers (HWs) is the gold standard, using a calcium alginate, rayon, Dacron, or nylon swab and STGG transport medium^25^. The “gold standard”, although highly effective in detecting pneumococcal carriage, has several disadvantages for community-based sample collection. It requires trained staff, which has both cost and logistical implications for bringing participants to clinics, or trained staff to participants’ homes. Collection of nasopharyngeal samples may also cause discomfort for participants. Nasal swabs, or nose blowing (if participants have visible nasal secretions) may be more feasible^26^. Few studies have directly compared those methods with the “gold standard”, but nasal swabs have been shown to have comparable sensitivity, and be cheaper and more widely accepted by the community^27–29^. However, two of those studies sampled children with symptoms of acute upper respiratory infection^27,28^, and the other, although it had a large population of healthy adults and children, was carried out in a high-income country^29^. There is insufficient evidence comparing sampling methods for *S. pneumoniae* carriage in healthy individuals on a population level in LMICs.

*Enterobacterales* are ubiquitous in the human digestive tract and can easily be recovered from faecal samples to test for colonisation with resistant bacteria. A previous community-based study in Vietnam used faecal samples collected by health-workers at health facilities^30^. Overall, 94% of isolates were resistant to at least one of the five antibiotics tested, and 60% were resistant to three or more antibiotics. Self-collected samples may be logistically easier, but the feasibility and acceptability of doing this on a large scale in community-based studies has not been explored.

We aimed to compare the feasibility, acceptability, bacterial recovery, and cost of HW-administered nasopharyngeal swabs and self-administered nasal swabs for recovery of *S. pneumoniae* in healthy adults and children, on a large scale, in a lower-middle-income setting, in Vietnam. We also aimed to explore the feasibility and acceptability of collection of faecal samples for culturing *Enterobacterales*.

## Methods

### Study design

We used a mixed-methods cross-sectional design, including household survey, specimen collection, and in-depth interviews, in order to evaluate the feasibility, acceptability, and bacterial recovery of different community-based swabbing and sample collection methods.

### Study population and participants

This study was conducted in the general resident population in one commune of Binh Luc district, Ha Nam province, northern Vietnam, between 16 July 2018 and 10 April 2019. The commune had a total population of approximately 9746 (2638 households), distributed across 22 villages and hamlets covering 8.7km^2^, and is a rural, predominantly farming area. Like 99% of communes in Vietnam, it has a commune health centre (CHC) staffed by a doctor/assistant doctor, several auxiliary staff including nurses/midwives and a pharmacist^31^. The CHC is approximately 9 km from the district hospital in Binh Luc, 12 km from the Provincial Hospital in Ha Nam, and 70 km from the National Institute of Hygiene and Epidemiology (NIHE) and Oxford University Clinical Research Unit (OUCRU) in Hanoi, where samples were processed.

**Figure 1:**
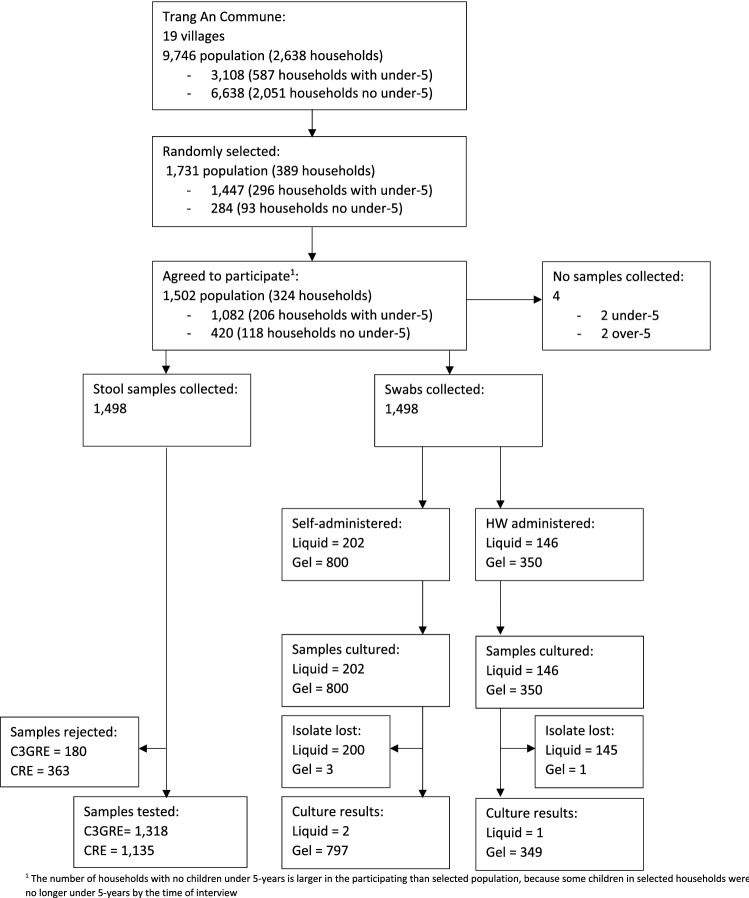
Flow diagram of participant inclusion.

A list of all households registered in the study area was obtained from the commune health centre and was used as the sampling frame. No further eligibility criteria were applied. Households with children under 5-years old were oversampled, in order to capture higher levels of pneumococcal carriage. 389 households were selected in total using the runiform command in Stata V14: 93 without children under 5-years, and 296 with children under 5-years old (Fig. 1).

Data from this study was expected to inform sample sizes for future larger community-based carriage studies, and estimates of many of the outcome indicators were not known. We assumed that 11% of the whole population would carry *S. pneumoniae* , based on a study in Vietnam that used a similar ratio of oversampling households with children under 5-years old^17^. There were three arms (see below), and a minimum sample of 499 per arm would be needed to detect a recovery rate 45% lower in one of the self-swabbing arms (i.e. 6.1% recovery) with 80% power. This represents a practically important difference. We randomly selected and recruited households until 500 participants were enrolled in each group. For all other prevalence estimates, using the most conservative value of 50%, we also reached the minimum of 385 per group to estimate with a margin of error of 5%, and a 95% confidence level. Carriage of *Enterobacterales* would be ubiquitous, and we would have sufficient precision to later estimate resistance among these isolates.

### Sample collection and laboratory analysis

Four health-workers from the local CHC were trained in data collection and nasopharyngeal swab collection. A list of selected households was given to health-workers, and potential participants were invited to participate. Health-workers visited households at an agreed time and collected written informed consent. Socio-demographic and behavioural information was collected by the health-workers, using structured questionnaires. All members of participating households were provided with containers and spoons for the collection of stool.

There were initially three swabbing groups: self-administered nasal swabs – Amies with charcoal gel-based transport medium with rigid cotton-tipped swab (Copan M40 414C Transystem™); self-administered nasal swabs – Amies without charcoal liquid transport medium with flexible minitip Nylon^®^ flocked swab (Copan eSwab™); and HW-administered nasopharyngeal swabs – Amies without charcoal liquid transport medium with flexible minitip Nylon^®^ flocked swab (Copan eSwab™) (Fig. 1). However, part way through the study we discovered a problem with the liquid transport medium, and the transport medium in those two arms was replaced with the Amies with charcoal transport medium (Copan M40 414C Transystem™) with a separate flocked swab (Fig. 1), so that there were now only two arms; self-administered nasal swabs and HW-administered nasopharyngeal swabs, both using Amies with charcoal.

Those in the self-swabbing group were provided with nasal swab kits (M40 414C Transystem™). Health-workers explained how to collect samples, and they provided a leaflet with diagrams explaining how to insert the swab about 2 cm into each nostril (anterior nares) and rotate 2–3 times, then place the swab into the tube until the swab reaches the end of the tube and seal (see Supplementary material). All households were provided with sample collection containers and leaflets for stool collection, and asked to store samples in a cool, dry place. Samples were collected from households the next day, stored in cool boxes with gel ice-packs during transit, and delivered to OUCRU and NIHE labs for immediate processing (within 24-h of collection). Those in the HW-administered nasopharyngeal swab group arranged a time for the health-worker to come back after the interview and collect swabs from all household members. The HW-administered swab tubes were taken back to the health centre and put directly into a cool-box with gel ice-packs and transported to the lab. Samples were cultured immediately on receipt in the laboratories. Due to the volume of samples, it was not feasible or cost-effective to store leftover specimens.

Standard microbiology culture and identification techniques were used to analyse the swab contents for the presence of *S. pneumoniae*^32^. Nasal/nasopharyngeal samples were cultured overnight at 37 °C in 5% CO_2_ atmosphere on 5% sheep blood agar supplemented with 5 mg/L of gentamicin to inhibit *Staphylococcus aureus*. Presumptive *S. pneumoniae* colonies were selected by colony morphology and α-haemolysis and subjected to optochin-susceptibility testing. MALDI-TOF (MBT Liberry 8468) to confirm the *S. pneumoniae* identification of optochin-susceptible colonies. Stool samples were cultured directly on two MacConkey agar No. 3 plates (CM0115 to inhibit enterococci growth) with 2 mg/L ceftazidime or 0.5 mg/L meropenem^33^. Plates were incubated overnight and large pink (lactose-fermenting) colonies were considered as positive for third-generation cephalosporin resistant *Enterobacterales* (C3GRE – proxy for ESBL) or CRE. Number of types of bacteria growing was based on the morphology.

### Statistical analysis

Bacterial recovery was assessed through comparison of recovery rates of *S. pneumoniae* between swabbing groups. Proportions of positive samples were calculated, with 95% confidence intervals. Recovery rates between swabbing and demographic groups were compared using logistic regression, with household as a random effect. Carriage rates are presented separately for children under 5-years and those aged 5-years and over. Feasibility was assessed through an internal review of the logistical procedures and storage, as well as qualitative interviews. We also calculated the cost per sample associated with each swabbing method, to allow cost comparisons. Costs were calculated as costs per household in each swabbing group and divided by the average number of household members. Costs were separated into swabs/swab packs, health-worker time costs, transportation, lab consumables, and lab staff time. Acceptability was assessed through refusal and withdrawal rates from the study logbook, as well as qualitative interviews. Results have been reported in accordance with guidelines for Strengthening the Reporting of Observational studies in Epidemiology (STROBE) (see STROBE checklist in supplementary files).

### Qualitative data collection and analysis

In-depth interviews with households and health-workers that participated in the survey, were conducted in August 2018. Each household participant interview was held within a week after samples were collected in order to learn about participant experiences and perspectives and to inform choices about measures and methods for future community-based sample collection. We planned to interview 3–6 study participants from each arm, purposively selected to represent a range of ages, family structures (i.e. with or without children under 5-years), and educational backgrounds. Saturation can develop fairly rapidly for studies where the aim is simply to understand common perceptions and experience among a group of relatively homogenous individuals, and we expected this small sample size to be sufficient^34^. We also planned to interview all four of the health-workers who conducted the fieldwork. Interviews explored participants’ and health-workers’ perceptions, experiences, and attitudes towards swabbing and sample collection, as well as how they stored the samples.

Thematic analysis was performed using a general inductive approach with NVivo 11^35^. Participants’ views were summarised according to the research questions about the feasibility, acceptability, and bacterial recovery for each method. Data were reviewed with the research areas in mind, but no *a priori* models were imposed. Lower order units of meaning were identified and clustered into themes. Within each theme, subtopics and different perspectives were looked for and then themes were reviewed for redundancy and to capture the essence of each category. Data were coded using these themes and quotes representing themes were selected and translated into English. Scripts were re-read at different stages of the analysis, to ensure that reporting remained true to the data.

### Ethics approval and consent to participate

The study was approved by Oxford Tropical Research Ethics Committee (Reference 552-17), the Institutional Review Board in Bio-medical Research of the National Institute of Hygiene & Epidemiology (Reference IRB-VN0105 7-01/2018) and the Ethics Committee of London School of Hygiene & Tropical Medicine (Reference 15831). Written informed consent was collected from all household members aged 18-years or older or parent/legal guardian of those under 18-years, and signed assent from those aged 12–18 years.

## Results

### Study population

We approached 389 households. In 62, the main respondent was not available for interview, and 3 refused, leaving 324 who agreed to participate, representing an 83.3% overall response rate. We collected data for 1502 individuals from these households. Faecal samples and nasal or nasopharyngeal swabs were collected from 1498 individuals (Fig. 1). 180 samples for C3GRE testing and 363 samples for CRE testing were rejected due to a lab error with growth media. Most nasal/nasopharyngeal samples transported in liquid medium were lost (345) due to batch failure, compared to only a few using gel-based medium^4^. Reference strains at different dilutions and delays also failed to grow in the liquid transport medium. Participant characteristics are shown in Table 1. Participants in each swabbing group were comparable, though slightly more stool and swab samples from children under 5-years, children with no education, and the poorest households were discarded, which may affect generalisability.

**Table 1:** Participant characteristics, logistical requirements, and costs.

		Total study population		Self-swabbing		Health-worker swabbing		Discarded swabs		Stool samples		Discarded stool samples	
		n	%	n	%	n	%	n	%	n	%	n	%
**Household characteristics**													
Number		324		170		75		79		281		43	
Members (median, range)		5	1–10	5	1–10	5	2–9	5	1–8	5	1–10	5	1–8
**Participant characteristics**													
Number		1498		799		350		349		1318		180	
**Age (years)**													
Under 5		246	(16.4)	126	(15.8)	57	(16.3)	63	(18.1)	211	(16.0)	35	(19.4)
5–9 years		158	(10.6)	83	(10.4)	41	(11.7)	34	(9.7)	143	(10.9)	15	(8.3)
10–19 years		131	(8.7)	68	(8.5)	33	(9.4)	30	(8.6)	118	(9.0)	13	(7.2)
20–29 years		241	(16.1)	137	(17.2)	48	(13.7)	56	(16.1)	200	(15.2)	41	(22.8)
30–39 years		270	(18.0)	149	(18.7)	70	(20.0)	51	(14.6)	240	(18.2)	30	(16.7)
40–49 years		118	(7.9)	59	(7.4)	28	(8.0)	31	(8.9)	108	(8.2)	10	(5.6)
50–59 years		170	(11.4)	94	(11.8)	28	(8.0)	48	(13.8)	159	(12.1)	11	(6.1)
60–69 years		105	(7.0)	57	(7.1)	29	(8.3)	19	(5.4)	85	(6.5)	20	(11.1)
70–100 years		59	(3.9)	26	(3.3)	16	(4.6)	17	(4.9)	54	(4.1)	5	(2.8)
**Sex**													
Female		776	(51.8)	416	(52.1)	174	(49.7)	186	(53.3)	685	(52.0)	91	(50.6)
Male		722	(48.2)	383	(47.9)	176	(50.3)	163	(46.7)	633	(48.0)	89	(49.4)
**Highest education**													
Children = < 16 years													
None		320	(21.4)	166	(20.8)	77	(22.0)	77	(22.1)	278	(21.1)	42	(23.3)
Primary		117	(7.8)	62	(7.8)	31	(8.9)	24	(6.9)	109	(8.3)	8	(4.4)
Secondary or higher		60	(4.0)	31	(3.9)	17	(4.9)	12	(3.4)	55	(4.2)	5	(2.8)
Adults > 16 years													
None		158	(10.5)	107	(13.4)	40	(11.4)	11	(3.2)	136	(10.3)	22	(12.2)
Primary		39	(2.6)	16	(2.0)	3	(0.9)	20	(5.7)	36	(2.7)	3	(1.7)
Secondary or higher		769	(51.3)	399	(49.9)	173	(49.4)	197	(56.4)	679	(51.5)	90	(50.0)
Missing		35	(2.3)	18	(2.3)	9	(2.6)	8	(2.3)	25	(1.9)	10	(5.6)
**Occupation**													
Farmer		495	(33.0)	253	(31.7)	96	(27.4)	146	(41.8)	444	(33.7)	51	(28.3)
Factory worker		176	(11.8)	102	(12.8)	44	(12.6)	30	(8.6)	151	(11.5)	25	(13.9)
Labourer		179	(12.0)	103	(12.9)	51	(14.6)	25	(7.2)	152	(11.5)	27	(15.0)
Other employment		65	(4.3)	36	(4.5)	14	(4.0)	15	(4.3)	56	(4.3)	9	(5.0)
Still in school		510	(34.1)	266	(33.3)	127	(36.3)	117	(33.5)	451	(34.2)	59	(32.8)
Not working		57	(3.8)	27	(3.4)	14	(4.0)	16	(4.6)	50	(3.8)	7	(3.9)
Missing		16	(1.1)	12	(1.5)	4	(1.1)	0	(0.0)	14	(1.1)	2	(1.1)
**Household wealth quintile**													
Poorest		307	(20.5)	179	(22.4)	57	(16.3)	71	(20.3)	263	(20.0)	44	(24.4)
Poor		306	(20.4)	151	(18.9)	70	(20.0)	85	(24.4)	287	(21.8)	19	(10.6)
Middle		289	(19.3)	184	(23.0)	78	(22.3)	27	(7.7)	239	(18.1)	50	(27.8)
Rich		304	(20.3)	137	(17.2)	88	(25.1)	79	(22.6)	263	(20.0)	41	(22.8)
Richest		292	(19.5)	148	(18.5)	57	(16.3)	87	(24.9)	266	(20.2)	26	(14.4)
**Feasibility**													
**Health-worker time spent per household (min)**													
Number of visits				2–3		2–6		NA		2–3		NA	
Travel^1^				50		80		NA		50		NA	
Consent				15		15		NA		15		NA	
Sample collection				0		40		NA		0		NA	
**Total time**													
Per household (min)				65		135		NA		65		NA	
Per sample (min)				14.1		29.3		NA		14.1		NA	
For 100 samples (hours)				23.5		48.8		NA		23.5		NA	
**Total samples required**													
For 100 positive samples				1000		730		NA		100		NA	
**Total time required**													
For 100 positive samples (hours)				235.0		356.5		NA		23.5		NA	
**Storage**													
Overnight storage place				Cool, dry place		Not stored overnight		NA		Cool, dry place		NA	
**Transportation to lab**													
N (%) within 24 h		1463/1494	(97.9)	789/796	(99.1)	343/348	(98.6)	NA		1459/1478	(98.7)	NA	
**Costs per participant/sample**													
Swabs/swab packs		NA		0.81		1.51		NA		0.4		NA	
**(USD $)**													
Health-worker time^2^		NA		0.62		1.29		NA		0.62		NA	
Transportation		NA		0.16		0.16		NA		0.16		NA	
Lab consumables		NA		3.83		3.83		NA		0.95		NA	
Lab staff		NA		1.84		1.84		NA		1.84		NA	
**Total costs**													
Per sample (USD $)		NA		7.26		8.63		NA		3.97		NA	
For 100 samples (USD $)		NA		726		863		NA		397		NA	
For 100 positive samples (USD $)		NA		7260		6300		NA		397		NA	

^1^Commune was 4 km at the widest point. Average travel time estimated at 10 min per household in each direction, and average of 2.5 visits per household for self-administered swabs and 4 visits for health-worker swabs.

^2^Based on health-worker time above, assuming 4.6 members per household and $2.64 per hour. Does not include interview time.

^3^$0.81 for the swab kit, plus $0.70 for a separate flocked swab.

Saturation for in-depth interviews was reached after six interviews with self-swabbing participants and three with HW-swabbing participants. Respondents were mainly farmers with secondary or high-school education, except one participant with college-level education. One respondent was male, eight were female , and family sizes ranged from one to seven persons. Three of the four health-workers involved in data collection agreed to be interviewed.

### Bacterial recovery and factors associated with *S. pneumoniae* carriage

A comparison of *S. pneumoniae* recovery for the different swabbing methods is shown in Table 2. The overall, pneumococcal recovery was 11.1% (128/1,149), with 10.0% (80/799) in self-administered swabs, and 13.7% (48/350) in HW-administered swabs (Table 2). For children under 5-years overall recovery was 26.2% (48/183), with 23.8% (30/126) from self-administered and 31.6% (18/57) from HW-administered swabs. Recovery from self-administered swabs (25.0%, 16/64) was similar to HW-administered swabs (21.9%, 7/32 ) for participants who had an ARI in the 2-weeks before the survey.

**Table 2:** Bacterial recovery for different swabbing groups.

Characteristic	Self-swabbing			Health-worker swabbing			Total recovery		
	n	N	%	n	N	%	n	N	%
Total	80	799	(10.0)	48	350	(13.7)	128	1149	(11.1)
**Age (years)**									
Under 5	30	126	(23.8)	18	57	(31.6)	48	183	(26.2)
5–9 years	17	83	(20.5)	8	41	(19.5)	25	124	(20.2)
10–19 years	4	68	(5.9)	4	33	(12.1)	8	101	(7.9)
20–29 years	4	137	(2.9)	2	48	(4.2)	6	185	(3.2)
30–39 years	11	149	(7.4)	10	70	(14.3)	21	219	(9.6)
40–49 years	1	59	(1.7)	1	28	(3.6)	2	87	(2.3)
50–59 years	5	94	(5.3)	1	28	(3.6)	6	122	(4.9)
60–100 years	8	83	(9.6)	4	45	(8.9)	12	128	(9.4)
**Sex**									
Female	39	416	(9.4)	23	174	(13.2)	62	590	(10.5)
Male	41	383	(10.7)	25	176	(14.2)	66	559	(11.8)
**Highest level of education—children = < 16 years**									
None	39	166	(23.5)	21	77	(27.3)	60	243	(24.7)
Primary	9	62	(14.5)	6	31	(19.4)	15	93	(16.1)
Secondary or higher	3	31	(9.7)	3	17	(17.7)	6	48	(12.5)
**Highest level of education—adults > 16 years**									
None	5	107	(4.7)	0	40	(0.0)	5	147	(3.4)
Primary	0	16	(0.0)	1	3	(33.3)	1	19	(5.3)
Secondary or higher	24	399	(6.0)	16	173	(9.3)	40	572	(7.0)
**Occupation**									
Farmer	14	253	(5.5)	9	96	(9.4)	23	349	(6.6)
Factory worker	8	102	(7.8)	5	44	(11.4)	13	146	(8.9)
Labourer	4	103	(3.9)	2	51	(3.9)	6	154	(3.9)
Other employment	1	36	(2.8)	1	14	(7.1)	2	50	(4.0)
Still in school	51	266	(19.2)	30	127	(23.6)	81	393	(20.6)
Not working	2	27	(7.4)	0	14	(0.0)	2	41	(4.9)
**Household wealth quintile**									
Poorest	16	179	(8.9)	4	57	(7.0)	20	236	(8.5)
Poor	22	151	(14.6)	9	70	(12.9)	31	221	(14.0)
Middle	12	184	(6.5)	14	78	(18.0)	26	262	(9.9)
Rich	13	137	(9.5)	15	88	(17.1)	28	225	(12.4)
Richest	17	148	(11.5)	6	57	(10.5)	23	205	(11.2)
**Time to lab**									
< 8-h	24	257	(9.3)	19	129	(14.7)	43	386	(11.1)
8 or more hours	56	541	(10.4)	28	220	(12.7)	84	761	(11.0)
**Sample period**									
Period1	14	257	(5.5)	4	34	(11.8)	18	291	(6.2)
Period2	27	243	(11.1)	18	130	(13.9)	45	373	(12.1)
Period3	39	299	(13.0)	25	185	(13.5)	64	484	(13.2)
**ARI in the last 2 weeks**									
No	61	687	(8.9)	41	315	(13.0)	102	1002	(10.2)
Yes	16	64	(25.0)	7	32	(21.9)	23	96	(24.0)

In unadjusted regression models, recovery was higher among those who were swabbed by health-workers (OR 2.06, 95%CI 1.07–3.96) (Table 3). All age-groups over 10-years had lower carriage than children under 5-years, while labourers and those not working had lower carriage than farmers. Consistent with the age effect, children who were in primary or secondary school had lower carriage than those who were not yet in school. There was higher carriage in Period 3 (spring) (13.2%) and Period 2 (autumn) (12.1%) than in Period 1 (summer) (6.2%), but this difference was only significant for children under 5-years. Having an ARI in the last 2-weeks (OR 3.13, 95% CI 1.38–7.11) and using antibiotics for ARI in the last two-weeks (OR 3.13, 95% CI 1.29–7.57) or last month (OR 2.07, 95% CI 1.08 3.96) were associated with higher carriage. After stratifying by age, health-worker swabbing, having an ARI in the last 2-weeks, and using antibiotics for ARI were no longer associated with higher carriage. There was no pneumococcal vaccination in this population.

**Table 3:** Factors associated with *S. pneumoniae* carriage.

Characteristic	Total recovery			Odds ratio			Odds ratio—children under 5-years			Odds ratio—5-years and older		
	n	N	%	OR	95%CI	*p* value	OR	95%CI	*p* value	OR	95%CI	*p* value
**Age (years)**												
Under 5	48	183	(26.2)	1								
5–9 years	25	124	(20.2)	0.85	(0.39–1.86)	0.683						
10–19 years	8	101	(7.9)	0.17	(0.05–0.61)	0.007						
20–29 years	6	185	(3.2)	0.06	(0.02–0.17)	<0.001						
30–39 years	21	219	(9.6)	0.23	(0.08–0.66)	0.007						
40–49 years	2	87	(2.3)	0.11	(0.02–0.57)	0.009						
50–59 years	6	122	(4.9)	0.29	(0.09–0.92)	0.035						
60–100 years	12	128	(9.4)	0.26	(0.09–0.71)	0.009						
**Sex**												
Female	62	590	(10.5)	1								
Male	66	559	(11.8)	1.15	(0.67–1.98)	0.621						
**Highest level of education—children = < 16 years**												
None	60	243	(24.7)	1								
Primary	15	93	(16.1)	0.67	(0.30–1.47)	0.315						
Secondary or higher	6	48	(12.5)	0.29	(0.07–1.26)	0.099						
**Highest level of education—adults > 16 years**												
None	5	147	(3.4)	1								
Primary	1	19	(5.3)	0.70	(0.07–7.17)	0.759						
Secondary or higher	40	572	(7.0)	4.45	(1.30–15.21)	0.018						
**Occupation**												
Farmer	23	349	(6.6)									
Factory worker	13	146	(8.9)	0.57	(0.21–1.53)	0.264						
Labourer	6	154	(3.9)	0.23	(0.08–0.66)	0.007						
Other employment	2	50	(4.0)	0.82	(0.13–5.37)	0.837						
Still in school	81	393	(20.6)	2.68	(1.15–6.24)	0.022						
Not working	2	41	(4.9)	0.20	(0.04–0.98)	0.048						
**Household wealth quintile**												
Poorest	20	236	(8.5)	1			1			1		
Poor	31	221	(14.0)	1.20	(0.42–3.37)	0.733	1.54	(0.32–7.53)	0.590	1.09	(0.36–3.35)	0.880
Middle	26	262	(9.9)	1.08	(0.37–3.14)	0.891	1.65	(0.40–6.80)	0.488	0.88	(0.26–3.05)	0.845
Rich	28	225	(12.4)	0.62	(0.22–1.70)	0.347	0.84	(0.22–3.15)	0.789	0.51	(0.16–1.68)	0.268
Richest	23	205	(11.2)	0.97	(0.35–2.73)	0.956	2.94	(0.68–12.64)	0.147	0.61	(0.16–2.29)	0.465
**Swabbing group**												
Self-administered	80	799	(10.0)	1			1			1		
HW-administered	48	350	(13.7)	2.06	(1.07–3.96)	0.031	2.14	(0.73–6.31)	0.165	2.05	(0.95–4.42)	0.066
**Time to lab**												
< 8-h	43	386	(11.1)	1			1			1		
8 or more hours	84	761	(11.0)	0.71	(0.38–1.34)	0.297	0.63	(0.19–2.06)	0.444	0.65	(0.31–1.37)	0.261
**Sample period**												
Period1	18	291	(6.2)	1			1			1		
Period2	45	373	(12.1)	1.89	(0.75–4.74)	0.176	3.43	(1.07–11.03)	0.039	1.55	(0.45–5.41)	0.486
Period3	64	484	(13.2)	1.85	(0.73–4.69)	0.197	1.51	(0.46–4.98)	0.493	2.05	(0.64–6.58)	0.226
**ARI in the last 2 weeks**												
No	102	1002	(10.2)	1			1			1		
Yes	23	96	(24.0)	3.13	(1.38–7.11)	0.007	2.23	(0.63–7.92)	0.215	2.63	(0.88–7.86)	0.084
**Antibiotic use for ARI in the last 2 weeks**												
No	106	1011	(10.5)	1			1			1		
Yes	19	87	(21.8)	3.13	(1.29–7.57)	0.012	1.98	(0.52–7.50)	0.314	2.74	(0.84–8.96)	0.096
**Any antibiotic use in the last month**												
No	91	928	(9.8)	1			1			1		
Yes	34	190	(17.9)	2.07	(1.08–3.96)	0.029	1.62	(0.57–4.56)	0.362	1.25	(0.43–3.59)	0.683
**Daycare attendance**												
No	26	108	(24.1)	1			1			1		
Yes	22	75	(29.3)	1.14	(0.41–3.13)	0.800	1.14	(0.41–3.13)	0.800	0.93	(0.44–1.98)	0.851
**Household has child under-5**												
No	23	307	(7.5)	1			1			1		
Yes	105	842	(12.5)	1.43	(0.69–2.97)	0.329	0.27	(0.16–0.45)	<0.001	0.93	(0.44–1.98)	0.851
**Crowding – more than 2 people per sleeping room**												
No	79	832	(9.5)	1			1			1		
Yes	49	317	(15.5)	2.05	(0.98–4.29)	0.056	3.06	(1.02–9.23)	0.047	1.57	(0.66–3.75)	0.308
**Indoor smoke**												
No	94	804	(11.7)	1			1			1		
Yes	34	345	(9.9)	1.35	(0.67–2.73)	0.404	2.42	(0.78–7.55)	0.125	1.26	(0.56–2.85)	0.570
**Handwashing facility with water and soap**												
No	13	121	(10.7)	1			1			1		
Yes	115	1028	(11.2)	0.93	(0.28–3.05)	0.906	2.52	(0.66–9.67)	0.177	0.74	(0.20–2.73)	0.646

### Bacterial recovery from stools

*Enterobacterales* were recovered from all samples that were tested. C3GRE were found in 1233 of 1318 (93.6%) tested stools samples and CRE were found in 17 out of 1135 (1.5%) tested stools samples. All samples containing CRE also contained C3GRE. Full details will be reported in a separate publication.

### Feasibility of sample collection

Five main themes related to feasibility emerged through the qualitative interviews: workload for data collectors; sample collection procedures; concerns about quality; storage and transportation; and disruptions (Supplementary Table 1).

#### Workload for data collectors

Health-workers visited each household at least two times: one to introduce the study, collect consent forms and conduct the interview, and one to collect samples and/or administer sample collection. For both swabbing groups, additional visits might be required to obtain consent forms from all participating household members (Table 1). For the HW-swabbing group, it could take up to 5–6 visits to finish sample collection. As working adults and schoolchildren were typically away from home during the day, health-workers might have to revisit late in the evening or very early in the morning to take their swabs. The estimated total amount of time health-workers spent on each household, including travel, gaining consent, and collecting samples, was 65 min for the two self-swabbing groups and 135 min for the HW-swabbing group. Time required per sample, per 100 samples and per 100 positive samples are shown in Table 1.

#### Sample collection procedures

All interviewees responded that the leaflet instructions were straightforward and easy to understand, particularly as the health-workers provided thorough explanations and reminders throughout the process. In the self-swabbing groups, it was not difficult for adults to perform the nasal swab, except for minor discomfort. With small children or elderly people, however, a few participants were less confident and asked health-workers to help. In practice, this meant that the health-worker offered instructions, but did not collect the sample themself. Swabbing children, even when performed by health-workers, was harder as some children got scared and refused to stay still. Other mothers did not have a problem swabbing their small children as they were used to similar tasks when taking care of the children’s daily health and hygiene. In the health-worker swabbing groups, some participants reported discomfort with the nasopharyngeal swabs.

Stool sampling proved to be a bigger issue for most interviewees. Disgust arose due to the smell and having to transfer stool from the paper bowl to the sample container. Health-workers found stool samples “very dirty” and “time-consuming” to collect. Timing was also a challenge, as participants had to ensure that the stool had been taken no more than 12 h before health-workers collected them, which was much more difficult to coordinate for young children and could therefore be delayed for several days.

#### Concerns about quality

Concerns about differences in quality between self-swabbing and HW-swabbing emerged. Health-workers felt that self-swabbing would reduce their workload, but they were concerned that self-taken swabs would be less reliable. They thought that community members might not swab properly, and do it ﻿“just like normal nose cleaning﻿”, leading to low-quality samples and low positive rates. This concern may have led to their willingness to swab participants in the self-swabbing groups if asked to. Though in practice this only happened on a few occasions.

#### Storage and transportation

Fieldwork was arranged to ensure that samples were transported to the lab as soon as possible after collection. Participants were instructed by health-workers to collect samples on the evening before the planned collection date, and store them in a cool, dry place overnight. All participants responded that they did this, however, this usually meant at room temperature, which ranged from 8 °C to 38 °C during the study period. They did not want to keep samples in their fridge due to fear of contaminating food from stool samples. Our laboratory logbooks showed that 97.9% (1,463/1,494) of samples arrived at the lab within 24-h of collection, in compliance with the manufacturer’s instructions^36^. The remaining 31 were within 48-h (Table 1).

#### Disruptions

We planned to conduct the survey and collect samples over a 2–3 month period, however there were some factors that disrupted field-work. Most community participants were engaged in some form of agriculture, and seasonal activities such as harvests kept them busy and difficult to meet at certain times. Health-workers also had competing priorities, with their own routine work to attend to. For example, vaccination campaigns stopped data collection for a week each month. We also stopped data collection for a few weeks while we investigated the problem with the liquid transport media.

#### Cost

Per swab, HW-administered swabs ($8.63) cost more than self-administered swabs ($7.26) to collect and culture, and this was mainly due to the cost of health-worker time, and the separate nasopharyngeal flocked swab (Table 1). However, given the higher bacterial recovery, the total cost required to obtain 100 samples positive for *S. pneumoniae* would be lower ($6300 for HW-administered compared to $7260 self-administered). Stool samples cost $3.97 to collect and culture, and $397 for 100 samples positive for *Enterobacterales*.

### Acceptability of sample collection

In general, participants were happy to take part, and confirmed their willingness to participate in a similar survey in the future. One elderly participant, having experienced pain with the HW-administered swab, was reluctant to take part again. Five main themes were identified related to motivation to participate in the study: sense of contribution; self-benefits; perceived effort of taking part; influence from others; and previous experience with health studies (Supplementary Table 2).

#### Sense of contribution

For some interviewees, while the study had no or little direct individual benefit, they were willing to take part for the community’s interest. One interviewee linked individual health to collective health, stating that any “epidemic” could not be suppressed by only one person. Health-workers said the financial benefit didn’t offset the workload, but they were doing this because it was their responsibility.

#### Perceived self-benefits

Some participants were motivated by the perceived benefits for themselves or their families, for example, they were glad to be made aware of a new health problem through the study. Some thought this was a free health check-up, and they would get results if they had any disease, reflecting a misunderstanding about the study’s objectives.

#### Perceived effort of participating

The perceived effort of taking part also influenced participation. Most interviewees found the level of effort acceptable. In the self-swabbing group, many participants stated that any adult could perform the swab and it was “mild”, “just a bit of discomfort”, or even “nothing to worry about”. Swabbing of small children was also “not so difficult”, especially when done by caregivers who were used to calming and persuading their children. By comparison, experiences with the HW-administered nasopharyngeal swab were described as “very painful” or “eye-watering”, and it could take several tries to swab children. However, since this was performed by health-workers, it was not a burden on participants. In contrast, the effort perceived by health-workers was much higher: the more work health-workers took on, the less the participants had to do and the less time it took them.

Despite the complaints about stool sampling, some interviewees thought it was fine because they had been provided with all the equipment to collect samples hygienically. The clarity of instructions and access to health-workers if they had further questions also made the process acceptable.

#### Influence from others

Some family members were initially reluctant to participate, but yielded to the insistence and persuasion of their wives/mothers/grandmothers, or the head of the household. One interviewee persuaded her children by saying that the local doctor’s family also provided samples for this study. People could be influenced to participate by their good relationship with and appreciation of the local health-workers. Participants mentioned frequently counting on health-workers for health advice, and their trusting relationship was apparent from the way they addressed or talked to one another. They also appreciated the health-workers for their effort and persistence during the survey and contribution to the community in general.

#### Previous experience with health studies

A few participants mentioned their previous experience of participating in health-related studies, which facilitated their understanding and motivated them to participate in this survey.

Two main themes emerged about reluctance to participate: disgust at the idea of sampling stool; and negative perceptions and issues in understanding the research (Supplementary Table 2).

#### Disgust at the idea of sampling stool

Most participants felt disgusted and awkward about collecting and handling stool. This was mentioned as a reason for initial reluctance rather than refusal to take part altogether.

#### Negative perceptions and issues in understanding the research

Unwillingness to participate was also linked to problems with understanding the purpose of the study or what the samples would be used for. Some interviewees reasoned that certain people were harder to convince because they had not heard the health-workers’ briefing or were not familiar with health activities. In some cases, the study was rejected or doubted due to a negative impression of research in general and aversion to the idea of becoming test subjects.

## Discussion

The aim of the study was to inform future research on bacterial carriage and antibiotic resistance at the population-level. We explored the feasibility, acceptability, and bacterial recovery for sample collection in the community, and compared different methods of swabbing. Recovery of *S. pneumoniae* was 11.1% overall, and 26.2% among children under 5-years. Recovery was higher for health-worker administered swabs (13.7%) than self-administered swabs (10.0%) (OR 2.06 (95% CI 1.07–3.96)). We found that it was feasible to collect swabs and stool samples in the community on a large scale. Issues that emerged for consideration in future studies were workload for data collectors, sample collection procedures, concerns about quality, storage and transportation, and disruptions due to agricultural work and competing health-worker priorities. The total cost of collecting and processing each sample was higher for health-worker collected swabs ($8.63) than for self-collected swabs ($7.26) and for stool ($3.97). But given the higher recovery rate, the total cost to obtain 100 positive swabs to use for subsequent susceptibility testing or sequencing would be less for HW-administered swabs ($6300) compared to self-administered swabs ($7260). We found that community members were willing to participate in the study, and only 3 households refused, indicating a high acceptability of taking part. Qualitative data highlighted themes related to sense of contribution, perceived self-benefits, perceived effort of participating, influence from others, and previous experience with health studies. Reasons for reluctance to participate included disgust with the idea of collecting stool samples, and negative perceptions of research. High recovery of third-generation cephalosporin resistant *Enterobacterales* (93.6%) is worrying, but validates the sampling procedures reported here. Future publications will report prevalence and determinants of resistance in *S. pneumoniae* and *Enterobacterales* in this population in more detail.

Overall, our results were similar to a previous pneumococcal carriage study in central Vietnam, in which doctors collected nasopharyngeal swabs from both healthy children and adults in a household survey in Nha Trang, October 2006^17^. They found an overall percentage of *S. pneumoniae* carriage of 11%. However, the carriage among children under 5-years was higher than ours at 43%. Other community-based studies in Vietnam also found higher carriage among children under 5-years, ranging from 28.7 to 52%^8,17–24^. However, most of these studies were conducted more than 10 years ago, before the rapid development and transition of Vietnam to a lower-middle income country. The most recent of these estimates, based on health-worker collected nasopharyngeal swabs in 2019, reported lower carriage of 28.7%^21^. A 2014 meta-analysis looking at carriage of *S. pneumoniae* in healthy children under 5-years old also found higher carriage in less developed countries, with a higher pooled prevalence in low-income countries (64.8%) than lower-middle income countries (47.8%)^16^. In a 2007 Vietnam-based study there were big differences in carriage between urban (26%), sub-urban (22%) and rural areas (60%). Together these findings suggest that the environment is important in determining *S. pneumoniae* carriage^22^.

Collection method is also important in determining bacterial recovery, and health-workers may better adhere to sample collection procedures and improve yield. But health-worker involvement also increases cost and time, and may be problematic in large-scale community studies. In many studies that use health-worker collected swabs, participants are invited to health facilities, or approached when presenting at antenatal or vaccination services. This reduces the burden on health-workers and allows samples to be plated immediately or refrigerated shortly after collection. However, in large-scale community surveys, inviting participants to come to the health centre can be complicated, may result in high refusal rates^17^, and potentially introduce biases. We collected samples during household visits, but this required health-workers to make many repeat visits to households, making this method more costly and time-consuming. To reduce the burden on sample collectors, we explored the feasibility of using self-collected samples. In our study, self-swabbing required fewer household visits and was generally found to be simple and easy to do for most participants. Comparison of health-worker swabbing and self-swabbing for COVID-19 and other respiratory pathogens shows that self-swabbing is acceptable and has comparable sensitivity and specificity^29,37,38^, though other reviews report lower sensitivity with self-swabbing from the same swab sites^39^.

Health-worker collected samples may also have resulted in higher bacterial recovery compared to self-collected samples due to higher pneumococcal density in the nasopharynx compared to anterior nares. There have been very few studies directly comparing nasal and nasopharyngeal swabs for detecting pneumococcal carriage in healthy people. A community survey in the UK found no significant differences in the recovery of *S. pneumoniae* between self-taken nasal swabs and HW-taken nasopharyngeal swabs^29^. A Canadian study found 95.2% sensitivity for nasal swabbing compared to nasopharyngeal swabbing for *S. pneumoniae* detection in children under 6-years, but only 52.5% sensitivity in those aged 6–15 years^40^. In our study recovery was lower or similar for self-swabbing in all age-groups. We know of no other studies in resource-limited settings that have compared these two methods. Most of the carriage studies in Vietnam used nasopharyngeal swabs^8,19^, while three older studies used nasal swabs^17,20,22^, so it is difficult to compare them directly. All were taken by trained healthcare workers, and recovery was similar (28.7–52% for nasopharyngeal swabs, and 35–49.4% for nasal swabs).

Another reason for higher recovery with HW-swabbing may be use of nylon flocked swabs rather than the cotton-tipped swabs used for self-swabbing. Nylon flocked swabs are more efficient than Dacron or rayon in recovering *S. pneumoniae* both in vivo and in vitro^41^. WHO expert working group recommends calcium alginate, rayon, Dacron, or nylon swabs, though experts also suggested that flocked swabs could improve sensitivity of detection, since they allow easier bacterial elution from swabs into STGG as well as higher yields of organisms^25^. There are no studies directly comparing yield with nylon flocked swabs and cotton-tipped swabs.

In the end, both arms of our study used the same transport medium, Amies with charcoal, whereas most previous studies in Vietnam have used STGG, the primary transport medium recommended by WHO. STGG is cheap, easy to make with ingredients that are commonly available^25^, and has been widely used in many carriage studies in different resourced settings with high recovery of *S. pneumoniae*^17,42,43^. There have been no field-based studies comparing STGG with other transport media to sustain *S. pneumoniae* viability at room temperature or cold storage, studies in either LMIC or high-income settings. Other studies have successfully used newer media with similar positive rates, including charcoal-based transport medium^8,19^, Amies^44^, or Amies with charcoal^29^. In this study, we used M40 Transystem™ Amies with charcoal media because it was available as a commercial swab kit and was easy to send to households for self-sampling.

Transportation time and storage procedures are other factors that might have affected *S. pneumoniae* viability and differed between groups. The WHO expert group recommends that the swab be inserted in a closed tube with STGG transport media, placed in a cool-box or on wet ice and transported to the laboratory within 8 h^25^. According to the suppliers, of the M40 Transystem swabs, they are stable for up to 24 h at room temperature (20–25 °C)^36^. Two other studies in Vietnam had swab transportation times up to 12 h using charcoal transport media at a cool temperature, and still had high recovery of pneumococci^8,19^. In a community-based survey in the UK, transportation took 1–2 days by post or taxi, with no refrigeration^29^. Although 97% of our samples arrived at the lab within 24-h, as recommended by the manufacturer, there was also a recommendation that samples be stored in a fridge or at room temperature during transit (20–25 °C)^36^. Some of our study took place during summer when average temperatures in the area were far higher than recommended, with the lower daily temperature around 26 °C and the higher over 30 °C^45^. Even during cooler spring and autumn months, maximum daytime temperatures were often above 25 °C. In particular, self-collected swabs were stored at room temperature overnight, and recovery during summer months for this group was particularly low (4.7%).

This is the largest community-based study to date comparing carriage rates and bacterial recovery of *S. pneumoniae* between self-swabbing and health-worker swabbing in a low- and middle-income country setting. This study provides important evidence for planning community-based carriage studies, including cost, logistics, and participant feedback about acceptability of different methods. The large, randomly-selected sample reduced possible selection biases, and data collectors were trained to reduce response bias. Lab analyses were conducted blind to the swabbing group. However, the limitations of this study include the loss of 349 samples, mainly in the liquid media groups, limiting the power of analyses looking at determinants of pneumococcal carriage. This also meant that we were unable to compare yield for the liquid and gel-based transport media. There were several methodological differences between self-swabbing and health-worker swabbing groups, including person who collected the sample, swab site, type of swab, and time to cold storage, and we don’t know which of these was responsible for the observed differences in bacterial recovery. Because we simultaneously collected swabs and stool samples, we are unable to say which placed more burden on data collectors in terms of number of return visits to households, and data collectors made multiple visits to households even in the self-swabbing arm in order to collect stool samples. Although we reached saturation, the small number of in-depth interviews, and the conduct of the study before self-swabbing for COVID-19 became widely used may also limit the generalisability of qualitative results.

## Conclusions

Sample collection for large community-based studies is feasible and acceptable to participants. Health-worker collected swabs were more costly and time-consuming to collect and caused some discomfort, but had higher pneumococcal recovery rates. Higher recovery means that the total cost for 100 positive samples was lower than self-collected swabs. Higher recovery from health-worker swabs may have been due to better adherence to the sampling procedure, nasopharyngeal sample, the flocked swab, the storage directly into a cool-box after collection, or a combination of these. Future studies will need to consider these trade-offs for population-based sample collection to estimate antibiotic resistance in commensal bacteria.

## Supplementary Information

Supplementary Information 1.

Supplementary Tables.

## Data Availability

Illustrative quotes form the qualitative data are provided in the supplementary tables. The quantitative datasets generated and analysed during the current study are not publicly available as this was not included in the consent process, but anonymised datasets can be made available from the corresponding author on reasonable request.

## References

[1] van Nguyen K, Thi Do NT, Chandna A, Nguyen TV, van Pham C, Doan PM (2013). Antibiotic use and resistance in emerging economies: a situation analysis for Viet Nam. BMC Public Health.

[2] Chua AQ, Verma M, Hsu LY, Legido-Quigley H (2021). An analysis of national action plans on antimicrobial resistance in Southeast Asia using a governance framework approach. Lancet Reg Health West Pac..

[3] Ministry of Health. National Action Plan on Combatting Drug Resistance in the period from 2013 to 2020. Hanoi (2013).

[4] Aabenhus, R., Jensen, J. U. S., Jørgensen, K. J., Hróbjartsson, A., Bjerrum. L. Biomarkers as point-of-care tests to guide prescription of antibiotics in patients with acute respiratory infections in primary care. *Cochrane Database Syst. Rev.* **2014**(11) (2014). 10.1002/14651858.CD010130.pub2 25374293

[5] McDonagh MS, Peterson K, Winthrop K, Cantor A, Lazur BH, Buckley DI (2018). Interventions to reduce inappropriate prescribing of antibiotics for acute respiratory tract infections: Summary and update of a systematic review. J. Int. Med. Res..

[6] Tonkin-Crine, S. K. G., Tan, P. S., van Hecke, O., Wang, K., Roberts, N. W., Mccullough, A. *et al*. Clinician-targeted interventions to influence antibiotic prescribing behaviour for acute respiratory infections in primary care: an overview of systematic reviews. *Cochrane Datab. Syst. Rev.* **9**(9) (2017). 10.1002/14651858.CD012252.pub2 PMC6483738 28881002

[7] Wei X, Zhang Z, Walley JD, Hicks JP, Zeng J, Deng S (2017). Effect of a training and educational intervention for physicians and caregivers on antibiotic prescribing for upper respiratory tract infections in children at primary care facilities in rural China: A cluster-randomised controlled trial. Lancet Glob. Health..

[8] Larsson M, Kronvall G, Chuc NTK, Karlsson I, Lager F, Hanh HD (2000). Antibiotic medication and bacterial resistance to antibiotics: A survey of children in a Vietnamese community. Trop. Med. Int. Health..

[9] Nga DTT, Chuc NTK, Hoa NP, Hoa NQ, Nguyen NTT, Loan HT (2014). Antibiotic sales in rural and urban pharmacies in northern Vietnam: An observational study. BMC Pharmacol. Toxicol..

[10] Do NTT, Vu HTL, Nguyen CTK, Punpuing S, Khan WA, Gyapong M (2021). Community-based antibiotic access and use in six low-income and middle-income countries: A mixed-method approach. Lancet Glob. Health..

[11] Costelloe C, Metcalfe C, Lovering A, Mant D, Hay AD (2010). Effect of antibiotic prescribing in primary care on antimicrobial resistance in individual patients: Systematic review and meta-analysis. BMJ.

[12] Bell BG, Schellevis F, Stobberingh E, Goossens H, Pringle M (2014). A systematic review and meta-analysis of the effects of antibiotic consumption on antibiotic resistance. BMC Infect. Dis..

[13] Goossens H, Ferech M, vander Stichele R, Elseviers M. (2005). Outpatient antibiotic use in Europe and association with resistance: a cross-national database study. Lancet.

[14] Guillemot D, Varon E, Bernède C, Weber P, Henriet L, Simon S (2005). Reduction of antibiotic use in the community reduces the rate of colonization with penicillin G-nonsusceptible Streptococcus pneumoniae. Clin. Infect. Dis..

[15] Andrejko K, Ratnasiri B, Hausdorff WP, Laxminarayan R, Lewnard JA (2021). Antimicrobial resistance in paediatric Streptococcus pneumoniae isolates amid global implementation of pneumococcal conjugate vaccines: A systematic review and meta-regression analysis. Lancet Microb..

[16] Adegbola RA, DeAntonio R, Hill PC, Roca A, Usuf E, Hoet B (2014). Carriage of Streptococcus pneumoniae and other respiratory bacterial pathogens in low and lower-middle income countries: A systematic review and meta-analysis. PLoS ONE.

[17] Talarico, C. A. Epidemiologic characteristics of colonizing streptococcus pneumoniae in Vietnam and implications for population vaccination [PhD]. The University of Michigan (2009).

[18] Larsson M, Nguyen HQ, Olson L, Tran TK, Nguyen TV, Nguyen CTK (2021). Multi-drug resistance in Streptococcus pneumoniae among children in rural Vietnam more than doubled from 1999 to 2014. Acta Paediatr..

[19] Hoa NQ, Trung NV, Larsson M, Eriksson B, Phuc HD, Chuc NTK (2010). Decreased Streptococcus pneumoniae susceptibility to oral antibiotics among children in rural Vietnam: A community study. BMC Infect. Dis..

[20] Parry CM, Diep TS, Wain J, Hoa NTT, Gainsborough M, Nga D (2000). Nasal carriage in Vietnamese children of Streptococcus pneumoniae resistant to multiple antimicrobial agents. Antimicrob. Agents Chemother..

[21] Nguyen HAT, Fujii H, Vu HTT, Parry CM, Dang AD, Ariyoshi K (2019). An alarmingly high nasal carriage rate of Streptococcus pneumoniae serotype 19F non-susceptible to multiple beta-lactam antimicrobials among Vietnamese children. BMC Infect. Dis..

[22] Schultsz C, Vien LM, Campbell JI, Chau NVV, Diep TS, Hoang NVM (2007). Changes in the nasal carriage of drug-resistant Streptococcus pneumoniae in urban and rural Vietnamese schoolchildren. Trans. R. Soc. Trop. Med. Hyg..

[23] Dhoubhadel BG, Yasunami M, Anh H, Nguyen T, Suzuki M, Vu TH (2014). Bacterial load of pneumococcal serotypes correlates with their prevalence and multiple serotypes is associated with acute respiratory infections among children less than 5 years of age. PLoS ONE.

[24] Lee NY, Song JH, Kim S, Peck KR, Ahn KM, Lee SI (2001). Carriage of antibiotic-resistant pneumococci among Asian children: A multinational surveillance by the Asian Network for Surveillance of Resistant Pathogens (ANSORP). Clin. Infect. Dis..

[25] Satzke C, Turner P, Virolainen-Julkunen A, Adrian PV, Antonio M, Hare KM (2013). Standard method for detecting upper respiratory carriage of Streptococcus pneumoniae: updated recommendations from the World Health Organization Pneumococcal Carriage Working Group. Vaccine..

[26] Leach AJ, Stubbs E, Hare K, Beissbarth J, Morris PS (2008). Comparison of nasal swabs with nose blowing for community-based pneumococcal surveillance of healthy children. J. Clin. Microbiol..

[27] Rapola S, Salo E, Kiiski P, Leinonen M, Takala AK (1997). Comparison of four different sampling methods for detecting pharyngeal carriage of Streptococcus pneumoniae and Haemophilus influenzae in children. J. Clin. Microbiol..

[28] Carville KS, Bowman JM, Lehmann D, Riley TV (2007). Comparison between nasal swabs and nasopharyngeal aspirates for, and effect of time in transit on, isolation of Streptococcus pneumoniae, Staphylococcus aureus, Haemophilus influenzae, and Moraxella catarrhalis. J. Clin. Microbiol..

[29] Coughtrie AL, Whittaker RN, Begum N, Anderson R, Tuck A, Faust SN (2014). Evaluation of swabbing methods for estimating the prevalence of bacterial carriage in the upper respiratory tract: A cross sectional study. BMJ Open.

[30] Dyar OJ, Hoa NQ, Trung NV, Phuc HD, Larsson M, Chuc NTK (2012). High prevalence of antibiotic resistance in commensal Escherichia coli among children in rural Vietnam. BMC Infect. Dis..

[31] WHO RO for the WP. Human resources for health country profiles: Viet Nam [Internet]. Available from: https://apps.who.int/iris/handle/10665/259990 (2016).

[32] Spellerberg B, Brandt C (2011). Streptococcus. Man. Clin. Microbiol..

[33] Wilson G, Mccabe D (2007). The use of antibiotic-containing agars for the isolation of extended-spectrum β-lactamase-producing organisms in intensive care units. Clin. Microbiol. Infect..

[34] Guest G, Bunce A, Johnson L (2006). How many interviews are enough? An experiment with data saturation and variability. Field Methods.

[35] Thomas DR (2006). A general inductive approach for analyzing qualitative evaluation data. Am. J. Eval..

[36] Copan. Transystem^TM^: Traditional Bacteriology Transport Swabs For Collection and Transport of Aerobic and Anaerobic Bacteria [Internet]. Available from: http://www.copanusa.com/products/collection-transport/m40-transystem/.

[37] Nguyen TT, Zeger WG, Wadman MC, Barksdale AN (2021). Accuracy and acceptance of a self-collection model for respiratory tract infection diagnostics: A concise clinical literature review. J. Emerg. Nurs..

[38] Seaman CP, Tran LTT, Cowling BJ, Sullivan SG (2019). Self-collected compared with professional-collected swabbing in the diagnosis of influenza in symptomatic individuals: A meta-analysis and assessment of validity. J. Clin. Virol..

[39] Tan SY, Tey HL, Lim ETH, Toh ST, Chan YH, Tan PT (2020). The accuracy of healthcare worker versus self collected (2-in-1) Oropharyngeal and Bilateral Mid-Turbinate (OPMT) swabs and saliva samples for SARS-CoV-2. PLoS ONE.

[40] Vuichard, D., Singh, P., Luinstra, K., Newton, J., Loeb, M., Smieja, M. Agreement between nasal midturbinate and nasopharyngeal swab to detect streptococcus pneumoniae by polymerase chain reaction in children and adults. *Open Forum Infect Dis.* 3(suppl_1) (2016).

[41] Dube FS, Kaba M, Whittaker E, Zar HJ, Nicol MP (2013). Detection of Streptococcus pneumoniae from Different Types of Nasopharyngeal Swabs in Children. PLoS ONE.

[42] Granat SM, Mia Z, Ollgren J, Herva E, Das M, Piirainen L (2007). Longitudinal study on pneumococcal carriage during the first year of life in Bangladesh. Pediatr. Infect Dis. J..

[43] Abdullahi O, Nyiro J, Lewa P, Slack M, Scott JAG (2008). The descriptive epidemiology of Streptococcus pneumoniae and Haemophilus influenzae nasopharyngeal carriage in children and adults in Kilifi district, Kenya. Pediatr. Infect Dis. J..

[44] Regev-Yochay G, Abullaish I, Malley R, Shainberg B, Varon M, Roytman Y (2012). Streptococcus pneumoniae carriage in the Gaza strip. PLoS ONE.

[45] World Bank Group. Climate Change Knowledge Portal [Internet]. [cited 2022 Mar 29]. Available from: https://climateknowledgeportal.worldbank.org/country/vietnam/climate-data-historical.

