# Supplementary material for: Feasibility, acceptability, and bacterial recovery for community-based sample collection to estimate antibiotic resistance in commensal gut and upper respiratory tract bacteria
Source: Sci Rep. 2022 Dec 29;12:22512. doi: 10.1038/s41598-022-27084-z (PMC9797900; doi:10.1038/s41598-022-27084-z)
Supplement: Supplementary file 2 — Supplementary Tables. [file 41598_2022_27084_MOESM2_ESM.docx]

**Supplementary Table 1: Qualitative findings related to feasibility of sample collection**

| **Theme** | **Illustrative quote** |
| --- | --- |
| **Workload for data collectors** | HW 1: If HWs perform all the swabs, it’s harder in the sense that we have to visit a lot of times. We have to collect their consents and explain or read instructions. In a household of 3 or 4, maybe only one person is home while the other 3 are at work, so we have to come back in the evening to collect samples. So we have to travel back and forth a lot.  Interviewer: So it’s less convenient.  HW 2: To sum up, each household takes 3-4 visits, that’s the minimum number.  (Health-workers)  “For us women with young kids, they only had to revisit once or twice, not too many times. Because they only had to revisit if samples were missing.”  (Female, family size of 5 with 2 children under 5-years, self-administered swab)  Interviewer: Do you remember how many times the HWs visited your family?  “Oh my god must be 5 to 6 times. First visit for signing. Then they visited to collect the stool or swab samples. Because during daytime the young was not at home, the HWs had to wait for them to return. Once they waited until 9 pm but they still had not come home. Then they had to visit again next evening.”  (Female, family size of 7 with 2 children under 5-years, HW-administered swab)  Interviewer: During the past 4 weeks when you collected samples, what time did you usually start work?  HW 1: 5.30 am.  HW 2: To collect samples, we worked harder than farmers. […] Urine samples could be collected on the afternoon before or when we took their consents. But we had to ensure that the stool samples met the time requirement. Do you know what we did? We came to people’s house in the morning when they were still sleeping. When they got up, we had to wait for them to exercise, brush their teeth and wash up. Some children couldn’t poop even after 2 days.  (Health-workers) |
| **Sample collection procedure** | **Clarity of instructions**  “I found [the leaflet] easy. There was nothing to it. Even the elders understood it and were able to follow. Any adult can understand and follow. We got it already by reading, but they also came to instruct us. It was mostly their instructions. To be honest, reading from the leaflet was less important, but we still did read it.”  (Female, farmer family size of 6 with 3 children, two children under 5-years) |
|  | **Nasal self-swabbing**  “We adults tried to collect our samples. To be honest, we had never inserted the swab that deeply into our nose. It smelled slightly pungent, and deep insertion was slightly uncomfortable, especially for kids. I only took samples from the small child; everyone else took their own samples. Swabbing was a bit uncomfortable, but it was easier than collecting the stool and urine samples.” (Female, retired teacher)  Participant: I’m their mother so I just told them to let me know when they pooped or peed. It was not hard at all. It was fine.  Interviewer: What about when you inserted the swab into their nose, was it uncomfortable?  Participant: Oh, I had to assure them. I said: “Be good and stay still. It’ll be over in a moment.” They were good and listened to their parents, so there was no problem.” (Female, farmer family size of 6 with 3 children, two children under 5-years)  **Health-worker swabbing**  “The swab was extremely uncomfortable. It was eye-watering. […] It was a bit harder with children. Adults would just feel uncomfortable, but it was really hard to swab the children and took quite a lot of time. […] This one was difficult. He kept vomiting so it took several tries to take his swab.” (Female, farmer, family size of 7) |
|  | **Stool sampling**  HW1: We already instructed them but they messed it around the container and the zip lock bag so we had to change the bag to make it less dirty before sending to you [**…**]  HW2: You would not know if you don’t do it directly. It was DIRTY, STINKY [**…**]  HW3: Some stool samples were too messed up that I had to change the container.  (Health-workers)  Participant: Well, it’s easier to take urine samples. But the smell of the stool put pressure on me.  Interviewer: Even after you put on a mask and gloves?  Participant: I put on two layers, not just one.  (Female, family size of 5 with 2 children under 5-years, self-administered swab)  “We managed to collect our own urine and stool samples, but the two kids didn’t poop that day so they had to wait. They had to visit a few times. Same thing for swabbing. It was so hard to perform on the older kid; the 2 health-workers tried several times without success. It took them 2-3 visits to collect his swab sample.”  (Female, family size of 7 with 2 children under 5-years, HW-administered swab) |
| **Quality** | “Self-swabbing would be more convenient but good quality is not guaranteed. So if you want your rate, your number, you could not achieve it.” (Health-worker)  “Think about it. When you tell people to swab their nose, they could think it is just like a normal cotton bud used for daily hygiene, they don’t think that swabs are taken to **…**to**…** achieve the goal of**…** of**…** to see if there are bacteria in themselves.” (Health-worker)  “How can we swab ourselves? We can’t guarantee the same quality as when health-workers do it. It’s more guaranteed if health-workers do it. If we did it, we would only reach the outer part because we would be afraid that it would hurt. Only health-workers can deeply insert the swab.”  (Female, family size of 7 with 2 children < 5 years old, HW-administered swab)  “My mother? [laugh] too old that they had to swab her [laugh] [**…**] Too old! She waited, waited for the health-worker to swab her! She was not sure about doing it. Then she had to wait for them to come swab her [**…**] She was not sure whether she could do it right.” (Female, family of 6 members with 2 children under 5-years, self-administered swab) |
| **Storage** | “Who would keep that in the fridge? I only stored it somewhere well-ventilated. […] How could we keep that in there with our food?” (Female, family size of 7 with 2 children under 5-years old, HW-administered swab)  “I just put it in the bathroom. I wrapped it up several times in a plastic bag and kept it neatly in there. […] Our bathroom is really well-ventilated.” (Female, family size of 5 with 2 children under 5-years, self-administered swab) |
| **Disruptions** | “In the countryside, when there is less farm work, it’s really easy. It rains a lot in the 7^th^ month so people don’t work their fields, they don’t have other work, and the workload at companies isn’t too much around this time. So we can put in extra efforts to collect 100 samples or more. Why the extra efforts? Because we can make an effort this week, but in the next 3 weeks, we won’t be able to. We’re trying to ensure the study’s progress, but these days, we can’t collect from any households and we might not even manage 50 samples per week because children have to go to school (Another voice: People have to harvest their crops), and we have our personal business and the health station’s work too.”  (Health-worker)  HW1: We’re trying to ensure the study’s progress, but these days, we can’t collect from any households and we might not even manage 50 samples per week because children have to go to school (Another voice: People have to harvest their crops), and we have our personal business and the health station’s work too.  HW2: In October I have more work at the health station too, because we have year’s end paperwork and auditing…  HW1: So this isn’t the only thing we have to do. It would be easy if we only had to do this! That’s why we’re trying hard. |

**Supplementary Table 2: Qualitative findings related to acceptability of sample collection**

| **Theme** | | **Illustrative quote** |
| --- | --- | --- |
| **Motivations to participate** | | |
| **Sense of contribution** | “If the community is healthy then we members are healthy. If there is an epidemic in this area, I could not suppress it by myself right? So we all will participate, we all will advocate, I myself could not go against the community.” (Male, farmer, 52 years old)  “I asked in your study whether I know my status. They explained that only general results would be sent to the health centre, not to individuals. Then I said in terms of society and community this is very good, it’s OK that I myself get nothing. I thought that if I could have my own result, that would be extremely good.” (Female, retired teacher)  “We have to do this because it’s our job. We are primary health-workers so we have to do this. Otherwise, if you were to hire me for the job, I would never do it, to be honest. […] I have to do this because it’s my responsibility.” (Health-worker) | |
| **Self-benefits** | “I have my samples tested**…** for examples at least I will have result, if it’s normal then I am fine, otherwise I will know if I need to get treatment.” (Female, farmer, family size of 5 with 3 children, one child under 5-years)  “When people know that this programme is good everyone will like it, same for everyone. You know about your illness [**…**] Normally who will spend money on health check-up... it’s really difficult [**…**] Cost nothing and we get some money. Also feel pleased.” (Female, farmer family size of 6 with 3 children, two children under 5-years)  “If you hadn’t brought this [study] here, I wouldn’t understand. By talking to you today, and to the health-workers the other day, I was able to understand, so I was eager to participate.” (Female, farmer family size of 6 with 3 children, two children under 5-years) | |
| **Perceived effort of taking part** | **Participants**  “Did she feel pain or cry when you took her swab?”  “No. I encouraged her then I did it gently, then it was ok [**…**] It was just like taking earwax, encouragement was enough, not too difficult.” (Female, retired teacher, self-administered swab)  “It smelled bitter after the swab. That was the only thing. Otherwise during the swabbing process I felt quite normal and easy.” (Female, farmer, self-administered swab, family size of 4, with 2 children < 5-years old)    “My mother could do it herself so there was no need for my help. I only needed to help the kids; the adults could do it without a problem. It was quite easy and simple to collect samples. There was nothing to it. Everything had been prepared for us. We only needed to collect a bit, and there were spoons and gloves for hygienic purposes. It would have been gross if we’d had to pick it up by hand, but it was fine because it was clean.” (Female, family of 6 members with 2 children < 5, self-administered swab)  “I found [sample collection] quite easy, no problem. I would feel reluctant if I didn’t know, but now that I’ve participated and I know how it is, there’s nothing to it.” (Female, farmer, family size of 4 with 1 children)  “[The health-worker] gave me the leaflet and it was quite easy to understand. Doctor H lives nearby and he guided me too, so that made it easier to participate.” (Female, farmer, family size of 4 with 1 children)  **Health-workers**  “ So far we’ve known our way around the village, but soon we will have to search for each household. There won’t be time for that. We have to go on duty [at the health station], we have children to take to school (HW2: There were parents who didn’t remember their children’s names, who had moved out of the household) and our other roles at the health station too. […] So to collect so many samples these past few weeks and exceed your requirements was a tremendous effort from our part. It definitely wasn’t easy.”  (Health-worker)  “We came to people’s house in the morning when they were still sleeping. When they got up, we had to wait for them to exercise, brush their teeth and wash up. Some children couldn’t poop even after 2 days. The working hours were worse than those of a manual worker [laughs]. My family even asked me what I was doing, heading out so early and returning so late. The first 3 days of the week, we had to do this in addition to our work at the health station. During the first week, I visited each family, checked each person, labelled the samples and checked again before bringing them here. And there were dozens of stool samples to collect at the time. I visited household A and their samples weren’t ready, then I went to household B but they only had urine samples, then household C was missing their nasal samples. It was exhausting. One such morning was like a workout session to me. I lost some weight.” (Health-worker) | |
| **Influence from others** | Family/peer influence:  “This time he said he would turn a blind eye to it, he said OK, he’d do it for me.”  (Female, family size of 5 with 3 children, one < 5 years old)  “I said to my daughter you are not allowed to refuse [**…**] I said do it, she said it is so gross, I said it is gross but you have to do it!” (Female, farmer family size of 7 with 2 children, two under 5-years)  “Yes, I am like a propagandist. My family is easy. I can persuade my family, no difficulties.” (Female, farmer, family size of 4 with 1 children)  Participant: Ah yes in my family I take full responsibility for everything.  Interviewer: Then if you make a decision everyone else will follow.  Participant: Yes [laugh] [**…**] If one still hesitates I will explain and instruct them until they fully understand. So they will all follow me.  (Male, 52 years old, family size of 6 with 1 child under 5-years)  “They asked me what this was for. I didn’t know either and I told them to just do it. I told them that Doctor H’s family was also doing it it, so it wasn’t just our family. They only stopped asking when I mentioned Doctor H” (Female, farmer, family size of 4 with 1 children) | |
|  | Relationship with/appreciation of health-workers:  “I live in this village so I can go to the health centre if I have questions.. I always go visit the health centre when someone gets sick..” (Female, retired teacher)  “Basically, communication activities are also... already well done in our commune, so it’s easy. [**…**] They’ve done it well. I have to acknowledge their work. So there’s no complaints about them. So we voluntary agreed to take part.” (Male, 52 years old, farmer)  Interviewer: Did they have to come here many times?  Participant: Oh my**…** at least five to six times. Millions of times. Many days it was bloody rainy, it rained cats and dogs, those two were so hard-working [**…**] They were do enthusiastic. You too. Only a little of**…** that sample**…** well**…** all for people’s health! (Female, farmer, family size of 7) | |
| **Past experience with health studies** | “I’m not being smug here but I’ve joined a lot of activities before. I’m familiar with the procedures so as soon as they introduced [this study], we understood everything.” (Male, 52 years old, family size of 6 with 1 child under 5-years)  “Well in this area if there is any study, they usually come to me. After interviewing with health- workers that morning, taking samples, there was also a Medicine School thing, well, in the afternoon, with some young men. There was also a woman, like a leader of **…** things **…** of vaccination for children, then she also guided her to me. Again, answered another set of questions!” (Female, farmer) | |
| **Reluctance to participate** | | |
| **Disgust at stool sampling** | “My husband complained a bit about taking stool and urine.” (Female, farmer, family size of 4 with 2 children)  “At first my kids did not want to. They said it felt disgusting, they would not do it, what the hell was taking stool and nasal swabs for, they would not do it.” (Female, farmer, family size of 4 with 1 children) | |
| **Negative perceptions and issues in understanding the research** | “At first my daughter-in-law was also scared**…** she said what if they used the samples to do something**…**” (Female, farmer, family size of 6 with 2 children)  “Firstly, the most difficult one was my husband, when he got home, I had to explain for so long before he agreed. [laugh] He said I was talking nonsense. I said this could not be nonsense you’d better do it. He said it was such nonsense! I said no it is not nonsense. […] Me, I already understood because I had heard [the briefing], but it took a lot of persuasion to swab him.” (Female, farmer, family size of 4 with 2 children)  “Some people don’t understand because they haven’t participated in the [health] centre’s public programmes. They aren’t well aware so they will object. As for us, once we have participated in community activities, it’s quite easy.” (Male, 52 years old, family size of 6 with 1 child under 5-years)  “Take my granddaughter for example. Each time the district holds that immunisation activity, some people here said that we shouldn’t bring her there because she would become a lab rat for experimenting. I said there was no such thing as experimenting.” (Male, 52 years old, family size of 6 with 1 child under 5-years)  “Some families understood that it was for the community, for society. But others were more narrow-minded, they didn’t see any benefit or result, there was nothing in it for them. They only thought they would become experimental subjects.” (Health-worker) | |
